# Cavernous hemangioma of the parotid gland

**DOI:** 10.4322/acr.2023.436

**Published:** 2023-06-12

**Authors:** Ravi Hari Phulware, Amrita Talwar, Arvind Ahuja

**Affiliations:** 1 All India Institute of Medical Sciences, Department of Pathology and Laboratory Medicine, Rishikesh, Uttarakhand, India; 2 ABVIMS, PGIMER & Dr Ram Manohar Lohia Hospital, Department of Pathology, New Delhi, India

**Keywords:** Hemangioma, Hemangioma, cavernous, Parotid gland, Parotid neoplasms, Parotid region

Cavernous hemangioma (CH), or cavernoma, is a type of benign tumor occurring mostly in the brain, liver, skin, and retina.^1,2^ Although less commonly, CH can also occur in the spine, orbit, gastrointestinal tract, skeletal muscle, and long bones.^1^ CH comprises a cluster of abnormally dilated blood vessels that form a mass or lesion.^2,3^

CH is more commonly found in women than men and typically occur between the ages of 40 and 60. Their precise incidence needs to be well-established; however, they account for 2-4% of all parotid gland tumors.^2^ Still, they are considered one of the less parotid gland common types of tumors.^2,3^ Most parotid gland tumors are benign, with only about 20% malignant.^1,2^

CH is typically diagnosed by imaging tests such as MRI or CT scans. The location, size, symptoms, and general health of the patient are among the variables that affect how cavernous hemangiomas are treated. Treatment options in symptomatic cases with functional impairment and high risk of bleeding, include medical (steroid or interferon), embolization, surgery, or radiosurgery.^2,4^

Parotid gland tumors can be either benign or malignant. CH of the parotid gland is a relatively rare but well-documented entity in the medical literature. There is currently not enough knowledge on the prevalence of CH in the parotid gland; there were roughly 50 cases reported worldwide, most of which were individual case reports. Overall, the literature suggests that cavernous hemangioma of the parotid gland is a rare benign entity more common in females and typically presents as a painless mass in the parotid gland area. Surgical removal is the primary treatment, and this entity has a good prognosis and a low recurrence risk.^1-4^ CH of the parotid gland can present with various symptoms, including a painless mass or swelling in the area of the gland, facial nerve weakness or paralysis, and even hearing loss in some cases. The diagnosis of a CH is usually based on imaging studies such as MRI or CT scans, which can show a well-defined, sharply demarcated mass with areas of low and high intensity.^2-4^

A hemangioma's histopathological appearance can help determine the appropriate management and treatment options. Capillary hemangiomas may be treated with topical or oral medications, while surgical removal may be necessary for cavernous hemangiomas, depending on their size and location.^2,3^

Treatment options for parotid gland cavernous hemangiomas depend on the lesion’s size and location, the patient's symptoms and overall health status. In some cases, surgical removal of the tumor may be necessary, which can be challenging due to the proximity of the facial nerve and the risk of its injury. In other cases, observation or radiation therapy may be recommended.^3-5^

Cavernous hemangioma is a type of vascular malformation, but other types of vascular malformations can present with similar symptoms or imaging findings. The differential diagnosis of vascular malformation with cavernous hemangioma includes (i) Venous malformation: a type of vascular malformation that affects veins, and it can look similar to cavernous hemangioma on imaging studies. However, venous malformations typically have a more uniform appearance, while cavernous hemangiomas have a characteristic “popcorn” appearance due to multiple blood-filled spaces; (ii) Capillary malformation: a type of vascular malformation that affects small blood vessels called capillaries. Capillary malformations can present as flat, red, or pink marks on the skin and can sometimes be mistaken for cavernous hemangioma; (iii) arteriovenous malformation: a type of vascular malformation that involves abnormal connections between arteries and veins. Arteriovenous malformations can cause symptoms such as headaches, seizures, and neurological deficits and can be mistaken for cavernous hemangioma on imaging studies; (iv) Lymphatic malformation: a type of vascular malformation that affects the lymphatic vessels and can cause swelling or abnormal growths. Lymphatic malformations can sometimes be mistaken for cavernous hemangioma in imaging studies.^4,5^

**Figure 1:**
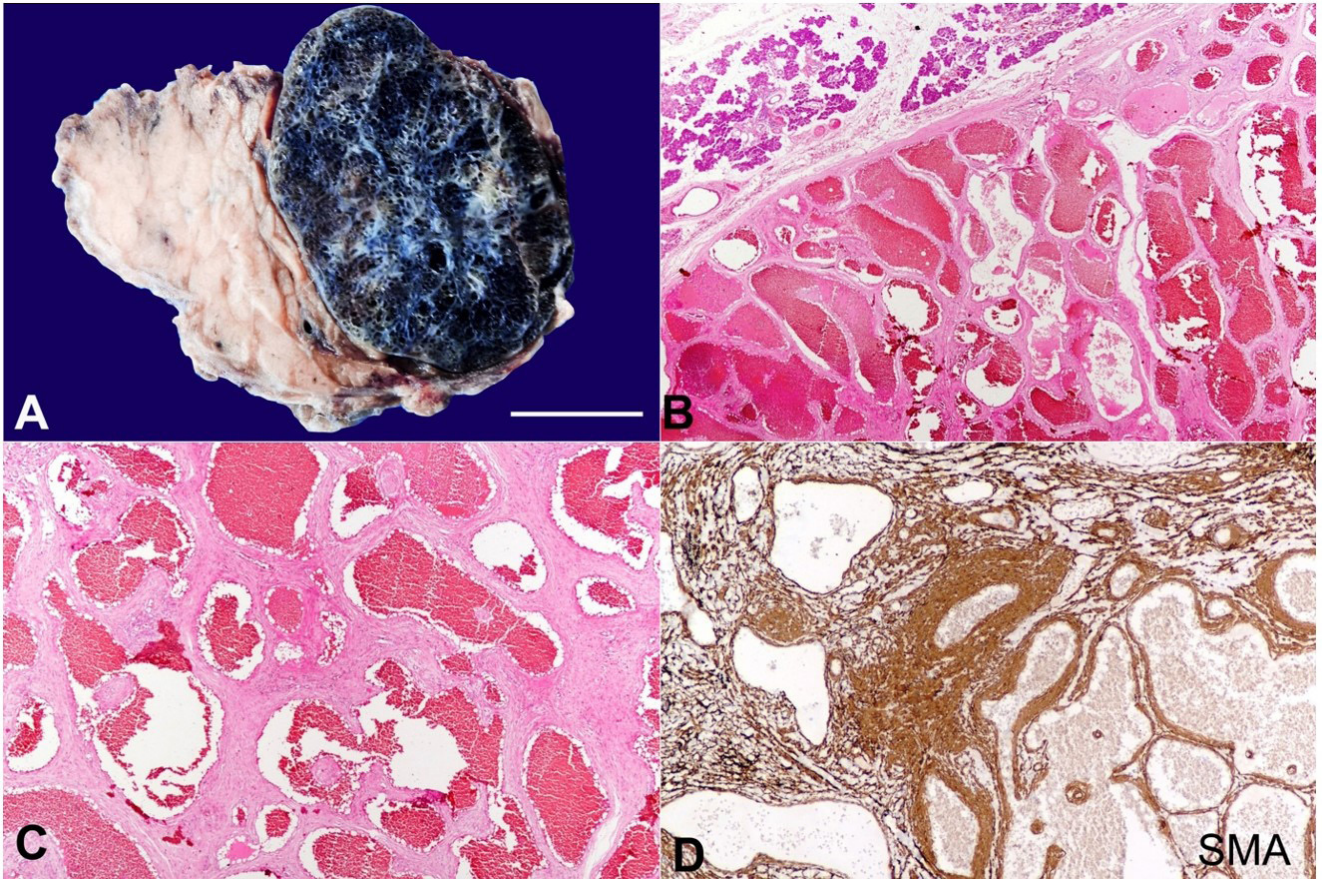
**A -** gross image showing normal salivary gland tissue along with a well-encapsulated tumor with areas of hemorrhage and comprising of numerous cystic spaces (vascular channels) scale bar = 2,5 cm; **B -** microscopic examination at low magnification showing normal salivary gland parenchyma along with a capsulated tumor comprised of many dilated vascular channels (H&E 100X); **C -** higher magnification image showing thick and thin walls vascular spaces in between fibro-muscular stroma (H&E, 400X); **D -** immunohistochemical reaction for smooth muscle actin (SMA) demonstrating smooth muscle bundles and vessel walls (SMA, 400X).

Figure 1 refers to a 42-year-old female patient with a painless, slowly growing mass in the right parotid region. The mass had been present for the past 6 months and has gradually increased. The patient reported no other significant symptoms, such as facial weakness or pain. The physical examination revealed a soft, non-tender mass in the right parotid region that measured approximately 6 cm. The overlying skin was normal, and there were no palpable lymph nodes in the neck. The ultrasound examination showed a well-circumscribed, hypoechoic lesion within the superficial lobe of the parotid gland, measuring 5.2 cm. The lesion had a cystic appearance with internal septations, consistent with a vascular lesion. The patient underwent an MRI, which confirmed the presence of a well-defined, lobulated mass in the superficial lobe of the right parotid gland, measuring 5.5 cm. The lesion was hyperintense on T2-weighted images and demonstrated heterogeneous enhancement with gadolinium. The imaging findings were consistent with a diagnosis of vascular malformation of the parotid gland. The patient was referred to a head and neck surgeon for further evaluation and treatment. Due to the lesion’s size and location, surgical resection was recommended. The patient underwent a superficial parotidectomy, and the postoperative course was uneventful. Histopathological examination of the resected specimen confirmed the diagnosis of CH (Figure 1A-1D). The patient was followed up for several months after the surgery, and there was no evidence of recurrence or complications.
